# On Deterministic and Stochastic Multiple Pathogen Epidemic Models

**DOI:** 10.3390/epidemiologia2030025

**Published:** 2021-08-12

**Authors:** Fernando Vadillo

**Affiliations:** Department of Mathematics, University of the Basque Country (UPV/EHU), Apdo 644, 48080 Bilbao, Spain; fernando.vadillo@ehu.es

**Keywords:** persistence time, epidemic models, stochastic differential equation, finite element method

## Abstract

In this paper, we consider a stochastic epidemic model with two pathogens. In order to analyze the coexistence of two pathogens, we compute numerically the expectation time until extinction (the mean persistence time), which satisfies a stationary partial differential equation with degenerate variable coefficients, related to backward Kolmogorov equation. I use the finite element method in order to solve this equation, and we implement it in FreeFem++. The main conclusion of this paper is that the deterministic and stochastic epidemic models differ considerably in predicting coexistence of the two diseases and in the extinction outcome of one of them. Now, the main challenge would be to find an explanation for this result.

## 1. Introduction

There have been numerous models for the spread of infectious diseases in populations. In the deterministic models, you may consult some excellent books on this subject, from the classic Chapter 10 in [1,2,3], and other excellent and complete references such as Chapters 9 and 10 in [4] and the monographs in [5,6]. All these deterministic models serve as a framework for formulating analogous stochastic models; these models are characterized by randomness and the variables are the solutions of stochastic differential equation (SDE). In this way, in my opinion there have been two ways to pass from the ODE to the SDE, one of them is simply adding new stochastic terms, for example, in [7,8,9,10]. The second one is explained in [11,12], and it is used in this paper. This technique begins by assuming different probabilities of the changes and calculating means and covariance matrix to get then a stochastic system. It has recently been shown in [13,14] that this difference in the stochastic part cause great differences in the asymptotic behavior of the solutions.

In this paper, we will consider a stochastic epidemic model with two pathogens. There are several studies on the evolution and the dynamics of deterministic epidemic models with multiple pathogens (see for example [15] and references therein). Later, the authors of [16,17] proposed a stochastic model, and their main conclusions are that both models differ considerably in predicting coexistence of the two pathogens, and the coexistence in the stochastic model has a very low probability. In this paper, we analyze the mean persistence time for this stochastic model solving numerically the backward Kolmogorov equation. In order to solve this equation numerically, we will use the Finite Element Method (FEM). This authors have studied this kind of problem in several papers [13,18,19] with very hopeful results to spread more complex problems.

The Stochastic Differential Equation (SDE) system for the dynamics of *n* variables has the form

(1) dX(t)=μ(t,X(t))dt+B(t,X(t))dW(t),

where $X={\left(X_{1},\cdots ,X_{n}\right)}^{T}$ and $W={\left(W_{1},\cdots ,W_{m}\right)}^{T}$ are *m* independent Wiener processes. The vectorial function μ(t,X(t)) is called the drift, and B(t,X(t)) is the diffusion matrix, a matrix n×m.

Obviously, a key question in epidemic models is to understand the constraints that lead to extinction or persistence of the disease. In order to study this question, let us define the random variable *T* that indicates the persistence time, i.e., the time it takes for the size of either variables to reach zero.

$$T\equiv \inf\{\;t\geq 0:X_{j}=0,\;\mathrm{for}\;\mathrm{some}\;j=1,\cdots ,n\;\},$$

obviously *T* depends on the initial value X(0) although it is not explicitly indicated.

As discussed in ([11], p. 150), the mean persistence-time τ≡E(T) for (1) satisfies the stationary backward Kolmogorov equation

(2) $$L\;\left(\tau \right)\;\equiv \;\sum_{j=1}^{n}\mu_{j}\;\frac{\partial \tau }{x_{j}}\;+\;\frac{1}{2}\;\sum_{j=1}^{n}\sum_{k=1}^{n}d_{jk}\;\frac{\partial^{2}\tau }{\partial x_{j}\partial x_{k}}\;=\;-1.$$

where $D=BB^{T}$ and with boundary conditions

(3) $$\left\{\begin{array}{c} \tau (\cdots ,x_{j-1},0,x_{j+1},\cdots )=0,\\ \frac{\partial \tau }{\partial x_{j}}\left(\cdots ,x_{j-1},M_{j},x_{j+1}\cdots \right)=0, \end{array}\right.$$

assuming that $x_{j}\leq M_{j},\;\;j=1,\cdots ,n$.

The Equation (2) is an elliptic partial differential equations of second order [20] or Chapter 8 in [21,22], really it is an advection–diffusion equation, and as the name suggests, the mean persistence time will depend on the operator D. These comments would explain the results in [13]. Moreover, in [7,8,9,10], the matrices D are diagonals which implies that the variables are not correlated, maybe an unrealistic hypothesis.

The paper is organized as follows. In Section 2.1, first we will present a stochastic epidemic model with two pathogens. Then, using the Symbolic Math Toolbox of Matlab©, we compute the equilibrium states for the deterministic part of the model and its numerical simulations by the classical Euler–Maruyama method. In Section 2.4, we will describe this techniques, based on the resolution of an associated backward Kolmogorov type equation for the mean persistence time, τ. In order to do the FreeFem++ implementation, we will first write a well-suited variational formulation and then we will present the numerical results. In Section 3, we compare the dynamics of the two models in tree examples, and finally in Section 4, we draw the main conclusions and some future researches.

Our numerical methods were implemented in Matlab© and FreeFem++, which are freely available and particularly efficient, see in [23]. The experiments were carried out in an Intel(R) Core(TM)i7-8665U CPU @ 1.90 GHz, 16.0 GB of RAM. The codes for the numerical tests are available on request.

## 2. Materials and Methods

### 2.1. Derivation of Stochastic Epidemic Model with Two Pathogen Strains

In this section, we will present an epidemic model with two pathogen strains and random demographics. The changes and their probabilities to the first order in ▵t are given in Table 1 with $x={\left(S,I_{1},I_{2}\right)}^{T}$. In this model, it is assumed that the infection with on strain immunizes for another disease.

Here, b>0 is the per capita birth rate and d(N)=1+0.05N is the per capita death rate, depending of the density $N\left(t\right)=S\left(t\right)+I_{1}\left(t\right)+I_{2}\left(t\right)$. Parameter $\beta_{j}>0$ is the transmission rate and $\alpha_{j}>0$ is the disease-related per capita death rate for individuals infected with strain $I_{j}$. Moreover, the per capital birth rate is divided into two parts: $b_{j}$ and $b-b_{j}$, if the strain *j* is passed from mother to offspring (vertical transmission), the per capital birth rate to the infected is $b-b_{j}$, in other case, if there is no vertical transmission, the newborn enter the susceptible class and $b_{j}=b$.

Fixing X(t) at time *t*, we calculate the expected change for the change $X={\left(S,I_{1},I_{2}\right)}^{T}$

(4) $$E\left(▵X\right)\;=\;\sum_{j=1}^{11}p_{j}\;▵X^{\left(j\right)}\;=\;\left(\begin{array}{c} \mu_{1}\left(S,I_{1},I_{2}\right)\\ \mu_{2}\left(S,I_{1},I_{2}\right)\\ \mu_{3}\left(S,I_{1},I_{2}\right) \end{array}\right)\;▵t,$$

where

(5) $$\left\{\begin{array}{c} \mu_{1}\left(S,I_{1},I_{2}\right)\;=\;S\left(b-d\left(N\right)-\frac{\beta_{1}I_{1}+\beta_{2}I_{2}}{N}\right)+b_{1}I_{1}+b_{2}I_{2},\\ \mu_{2}\left(S,I_{1},I_{2}\right)\;=I_{1}\left(b-b_{1}-d\left(N\right)-\alpha_{1}+\frac{\beta_{1}S}{N}\right),\\ \mu_{3}\left(S,I_{1},I_{2}\right)\;=\;I_{2}\left(b-b_{2}-d\left(N\right)-\alpha_{2}+\frac{\beta_{2}S}{N}\right). \end{array}\right.$$

and the covariance matrix

(6) $$E\left(▵X{\left(▵X\right)}^{T}\right)\;=\;\sum_{j=1}^{11}p_{j}\;\left(▵X^{\left(j\right)}\right){\left(▵X^{\left(j\right)}\right)}^{T}\;=\;D\left(S,I_{1},I_{2}\right)\;▵t,$$

where $D=(d_{i,j})$ is the diffusion matrix, a matrix 3×3 with $d_{2,3}=d_{3,2}=0$ and

$$\begin{array}{cc} d_{1,1} & =S\left(b+d\left(N\right)+\frac{\beta_{1}I_{1}+\beta_{2}I_{2}}{N}\right)+b_{1}I_{1}+b_{2}I_{2},\\ d_{j+1,j+1} & =I_{j}\left(b+b_{j}+d\left(N\right)+\alpha_{j}+\frac{\beta_{j}S}{N}\right),\;j=1,2,\\ d_{1,j+1} & =d_{j+1,1}=-b_{j}I_{j}-\beta_{j}\frac{SI_{j}}{N},\;j=1,2. \end{array}$$

Finally, the stochastic differential system (**SDS**) is

(7) $$dX\left(t\right)=\mu \left(t,X\left(t\right)\right)\;dt\;+\;D^{1/2}\left(t,X\left(t\right)\right)\;dW\left(t\right).$$

### 2.2. The Deterministic Model

The deterministic part is the following ordinary differential system (**ODEs**)q:

(8) $$\left\{\begin{array}{c} \frac{dS}{dt}\;=\;\mu_{1}\left(S,I_{1},I_{2}\right),\\ \frac{dI_{1}}{dt}\;=\;\mu_{2}\left(S,I_{1},I_{2}\right),\\ \frac{dI_{2}}{dt}\;=\;\mu_{3}\left(S,I_{1},I_{2}\right). \end{array}\right.$$

There are numerous theoretical studies on the evolution, persistence, or extinction of multiple pathogen strains for the deterministic epidemic models, see, for example, in [16,17] and their references. It has been proved that the dynamics of a deterministic model depends on the basic reproduction numbers defined by

$$R_{j}=\frac{\beta_{j}+b-b_{j}}{b+\alpha_{j}},\;j=1,2,$$

and it is known that if $R_{j}<1$, then the disease extinction occurs. Using the Symbolic Math Toolbox of Matlab©, we can compute six equilibrium values of the deterministic model which can be found in the Table 2, each column of this table is a zero of (8). We have denoted by ∗ nonzero values, whose exact formulas we did not write for their long expressions and lack of interest. Obviously, the asymptotic behavior of each equilibrium depends on the eigenvalues of the Jacobian.

### 2.3. Simulation Using the Euler–Maruyama Method

The numerical simulation for the stochastic differential system (7) implements the classic Euler–Maruyama numerical method, although it has strong order 1/2 and weak order 1 (see for example [24] or ([25] and more recently [26]). This method is simple and straightforward to implement [27].

We have written Euler–Maruyama algorithm, similar to an algorithm from in [18,19], with three stopping test, one for each of the variables *S*, $I_{1}$, and $I_{2}$, and with ▵t the time step. Essentially, given ▵t, the number of simulation and an initial position $\left(S\left(0\right),I_{1}\left(0\right),I_{2}\left(0\right)\right)$, the algorithm is straightforward until one of the variable is less than one, i.e., $S_{n}<1$ or ${\left(I_{1}\right)}_{n}<1$ or ${\left(I_{2}\right)}_{n}<1$. After all the trials, we computed the mean and standard deviation of stopping times. Matlab implementation is very similar to the program offered in the appendix of [18]. It is important to remark that in this implementation, at each step we have to compute the matrix $D^{1/2}$ using the Matlab’ command sqrtm.

### 2.4. The Mean Persistence Time for the Model

In this section, in order to predict the behavior of two pathogens in the stochastic model (7), we present an alternative technique based on the estimation of the mean existence time τ, by numerical resolution of the Kolmogorov type equation using the FEM and the FreeFem++ implementation.

Let us denote by s=S(0), $y_{1}=I_{1}\left(0\right)$, $y_{2}=I_{2}\left(0\right)$ and $\Omega =\left[0,M_{s}\right]\times \left[0,M_{1}\right]\times \left[0,M_{2}\right]$, where $M_{s},M_{1}$ and $M_{2}$ are positive constants and its boundary by $\partial \Omega =\Gamma_{1}\cup \Gamma_{2}\cup \Gamma_{3}\cup \Gamma_{4}$, where

$$\left\{\begin{array}{c} \Gamma_{1}=\left\{\left(s,y_{1},y_{2}\right)\in \Omega \;|\;s=0,\;\mathrm{or}\;y_{1}=0,\;\mathrm{or}\;y_{2}=0\right\},\\ \Gamma_{2}=\left\{\left(s,y_{1},y_{2}\right)\in \Omega \;|\;s=M_{s}\right\},\\ \Gamma_{3}=\left\{\left(s,y_{1},y_{2}\right)\in \Omega \;|\;y_{1}=M_{1}\right\},\\ \Gamma_{4}=\left\{\left(s,y_{1},y_{2}\right)\in \Omega \;|\;y_{2}=M_{2}\right\}. \end{array}\right.$$

The mean persistence time $\tau =\tau (s,y_{1},y_{2})$ for the stochastic model (7) satisfies the following stationary partial differential equation of Kolmogorov type:

(9) μ1(s,y1,y2)∂τ∂s+∑j=12μj+1(s,y1,y2)∂τ∂yjμ(s,y1,y2)+12d1,1∂2τ∂s2+12∑j=12dj+1,j+1∂2τ∂yj2+∑j=12d1,j+1∂2τ∂s∂yj=−1inΩ

with the following boundary conditions:

(10) $$\left\{\begin{array}{c} \tau \left(0,y_{1},y_{2}\right)=\tau \left(s,0,y_{2}\right)=\tau \left(s,y_{1},0\right)=0\;\mathrm{on}\;\Gamma_{1},\\ \frac{\partial \tau }{\partial s}\left(M_{s},y_{1},y_{2}\right)=0\;\mathrm{on}\;\Gamma_{2},\\ \frac{\partial \tau }{\partial y_{1}}\left(s,M_{1},y_{2}\right)=0\;\mathrm{on}\;\Gamma_{3},\\ \frac{\partial \tau }{\partial y_{2}}\left(s,y_{1},M_{2}\right)=0\;\mathrm{on}\;\Gamma_{4}. \end{array}\right.$$

As we said at the beginning of the paper, in order to preform the FreeFem++ implementation, we have to write a variational formulation for the boundary value problem (9) and (10). The next subsection will be devoted to do this.

### 2.5. Variational Formulation

In order to perform numerical experiments using FreeFem++, which is a partial differential equation solver and has its own language, we will write (9) and (10) in variational form. For this, we will make formal computations. More precisely, let us write (9) as follows:

(11) $$\begin{array}{cc} -\mu_{1}\frac{\partial \tau }{\partial s}-\mu_{2}\frac{\partial \tau }{\partial y_{1}} & -\mu_{3}\frac{\partial \tau }{\partial y_{2}}-\frac{1}{2}d_{1,1}\frac{\partial^{2}\tau }{\partial s^{2}}-\frac{1}{2}d_{2,2}\frac{\partial^{2}\tau }{\partial y_{1}^{2}}\\ \; & -\frac{1}{2}d_{3,3}\frac{\partial \tau }{\partial^{2}y_{2}^{2}}-d_{1,2}\frac{\partial^{2}\tau }{\partial s\partial y_{1}}-d_{1,3}\frac{\partial^{2}\tau }{\partial s\partial y_{2}}=1\;\mathrm{in}\;\Omega . \end{array}$$

Let us multiply multiple (11) by a regular “test” function $\phi (s,y_{1},y_{2})$ satisfying the homogeneous Dirichlet boundary conditions on $\Gamma_{1}$. Integrating over the domain Ω, the following terms with will appear:

$$\begin{array}{ccc} -\frac{1}{2}\int_{\Omega }d_{1,1}\frac{\partial^{2}\tau }{\partial s^{2}}\phi & = & \frac{1}{2}\int_{\Omega }d_{1,1}\frac{\partial \tau }{\partial s}\frac{\partial \phi }{\partial s}+\frac{1}{2}\int_{\Omega }\frac{\partial d_{1,1}}{\partial s}\frac{\partial \tau }{\partial s}\phi -\frac{1}{2}\int_{\partial \Omega }d_{1,1}\phi \left(\frac{\partial \tau }{\partial s}n_{s}\right),\\ -\frac{1}{2}\int_{\Omega }d_{2,2}\frac{\partial^{2}\tau }{\partial y_{1}^{2}}\phi & = & \frac{1}{2}\int_{\Omega }d_{2,2}\frac{\partial \tau }{\partial y_{1}}\frac{\partial \phi }{\partial y_{1}}+\frac{1}{2}\int_{\Omega }\frac{\partial d_{2,2}}{\partial y_{1}}\frac{\partial \tau }{\partial y_{1}}\phi -\frac{1}{2}\int_{\partial \Omega }d_{2,2}\phi \left(\frac{\partial \tau }{\partial y_{1}}n_{y_{1}}\right),\\ -\frac{1}{2}\int_{\Omega }d_{3,3}\frac{\partial^{2}\tau }{\partial y_{2}^{2}}\phi & = & \frac{1}{2}\int_{\Omega }d_{3,3}\frac{\partial \tau }{\partial y_{2}}\frac{\partial \phi }{\partial y_{2}}+\frac{1}{2}\int_{\Omega }\frac{\partial d_{3,3}}{\partial y_{2}}\frac{\partial \tau }{\partial y_{2}}\phi -\frac{1}{2}\int_{\partial \Omega }d_{3,3}\phi \left(\frac{\partial \tau }{\partial y_{2}}n_{y_{2}}\right),\\ -\int_{\Omega }d_{1,2}\frac{\partial^{2}\tau }{\partial s\partial y_{1}}\phi & = & \int_{\Omega }d_{1,2}\frac{\partial \tau }{\partial y_{1}}\frac{\partial \phi }{\partial s}+\int_{\Omega }\frac{\partial d_{1,2}}{\partial s}\frac{\partial \tau }{\partial y_{1}}\phi -\int_{\partial \Omega }d_{1,2}\phi \left(\frac{\partial \tau }{\partial y_{1}}n_{s}\right),\\ -\int_{\Omega }d_{1,3}\frac{\partial^{2}\tau }{\partial s\partial y_{2}}\phi & = & \int_{\Omega }d_{1,3}\frac{\partial \tau }{\partial y_{2}}\frac{\partial \phi }{\partial s}+\int_{\Omega }\frac{\partial d_{1,3}}{\partial s}\frac{\partial \tau }{\partial y_{2}}\phi -\int_{\partial \Omega }d_{1,3}\phi \left(\frac{\partial \tau }{\partial y_{2}}n_{s}\right), \end{array}$$

where the boundary terms are of the following form:

$$\left\{\begin{array}{ccc} \int_{\partial \Omega }d_{1,1}\phi \left(\frac{\partial \tau }{\partial s}n_{s}\right)\; & = & \int_{\partial \Omega }d_{2,2}\phi \left(\frac{\partial \tau }{\partial y_{1}}n_{y_{1}}\right)=\int_{\partial \Omega }d_{3,3}\phi \left(\frac{\partial \tau }{\partial y_{2}}n_{y_{2}}\right)=0,\\ \int_{\partial \Omega }d_{1,2}\phi \left(\frac{\partial \tau }{\partial y_{1}}n_{s}\right) & = & \int_{\Gamma_{2}}d_{1,2}\phi \frac{\partial \tau }{\partial y_{1}},\\ \int_{\partial \Omega }d_{1,3}\phi \left(\frac{\partial \tau }{\partial y_{2}}n_{s}\right) & = & \int_{\Gamma_{2}}d_{1,3}\phi \frac{\partial \tau }{\partial y_{2}}, \end{array}\right.$$

with $n_{s}$, $n_{y_{1}}$ and $n_{y_{2}}$ are the normal vectors exterior to the boundary $\Gamma_{2}$, $\Gamma_{3}$ and $\Gamma_{4}$, respectively.

## 3. Numerical Results

We present three numerical examples for the deterministic and stochastic epidemic models, it is remarkable that in Examples 2 and 3 the coexistence dynamics differ between the deterministic and stochastic models.

**Example** **1.** *In this first example, the per capital death and birth rates are* d(N)=1+N/100 *and* b=2 *. In addition,* $\beta_{1}=7,\beta_{2}=5.25,\alpha_{1}=\alpha_{2}=0.5$ *and* $b_{1}=b_{2}=3$ *, so that* $R_{1}=2;R_{2}=1.5$ *and according Theorem 2 in [16] the solutions to deterministic model (8) convergent to*

$$\lim_{t\to \infty }I_{1}\left(t\right)\;=\;39.35,\;\lim_{t\to \infty }I_{2}\left(t\right)\;=\;0.$$

**Figure 1:**
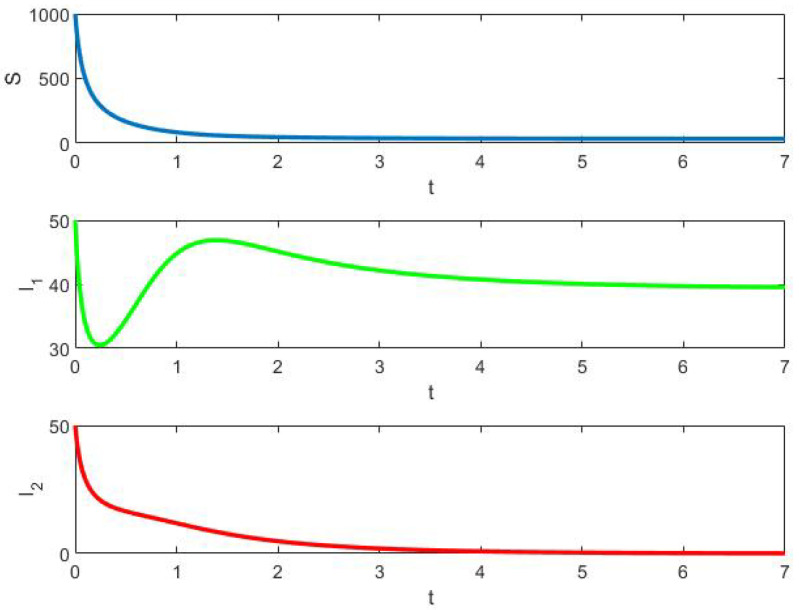
Example 1: deterministic solution for $S\left(0\right)=1000,I_{1}\left(0\right)=I_{2}\left(0\right)=50$.

*In Figure 1, we have plotted the solution of (8) using* Matlab *’command ode45 for $S\left(0\right)=1000,I_{1}\left(0\right)=50$, and $I_{2}\left(0\right)=50$ in 0≤t≤7. The final solutions are S(7)≈33.9912, $I_{1}\left(7\right)\approx 39.5387$, and $I_{2}\left(7\right)\approx 0.0712$.*

**Figure 2:**
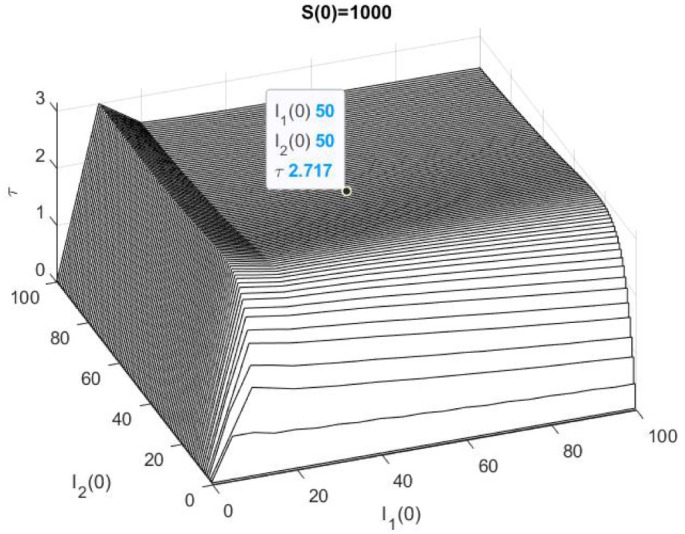
Example 1: S(0)=1000.

On the other hand, in Figure 2 we have represented the numerical solution of the problem (9) and (10) with $M_{s}=1000,M_{1}=M_{2}=100$. More precisely, we have represented $\tau (1000,I_{1}\left(0\right),I_{2}\left(0\right)$ for $0\leq I_{1}\left(0\right),I_{2}\left(0\right)\leq 100$, also we have highlighted that τ(1000,50,50)≈2.717.

In Table 3, we show the results of 10,000 trials of the Euler–Maruyama method with $▵t=10^{-4}$, and the value is represented in Figure 2. For each initial population size, we have written down the number of stops when S<1 or $I_{1}<1$ or $I_{2}<1$, and we have computed the mean stop time (mean) and its standard deviation (std). This result, which is close to the figure’s estimate, and it does not seem too far from the deterministic model, to get an idea in the deterministic problem $S\left(3\right)\approx 38.87,I_{1}\left(3\right)\approx 42.12,I_{2}\left(3\right)\approx 1.987$.

**Example** **2.** **Vertical transmission of both strains.***Let us suppose now that both strains are transmitted vertically. This corresponds to take*$b_{1}=b_{2}=0$ and b=6, $\beta_{1}=15,\beta_{2}=1$, $\alpha_{1}=2.5,\alpha_{2}=2$*. In this case, the basic reproduction numbers are*

$$R_{1}=2.4706\;>\;1,\;R_{2}=0.8750\;<\;1.$$

The equilibrium point is E=(2.1684,4.3367,54.2092), and the eigenvalues of the Jacobian are $-10^{-5}\pm 0.6682i$ and −3.0357. This means that the deterministic solution cycles closer and closer to each equilibriums as we can see in Figure 5 from [17], however its size fluctuates greatly.

**Figure 3:**
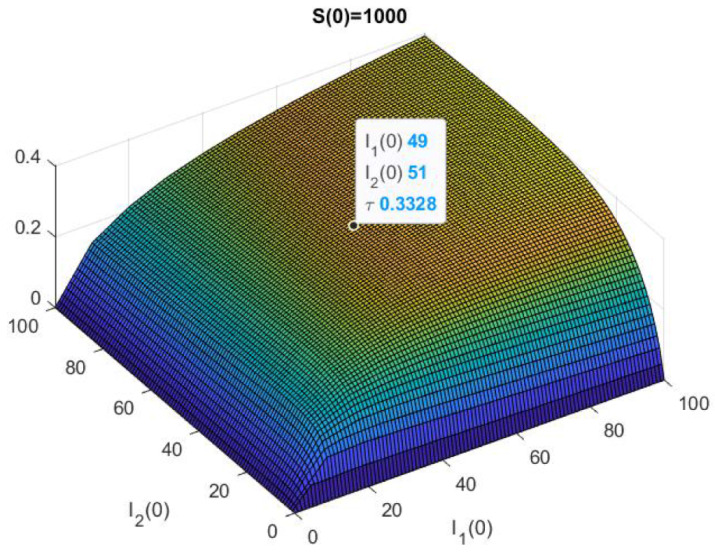
Example 2, vertical transmission of both strains: S(0)=1000.

In Figure 3, we have represented the numerical solution of the problem (9) and (10) with $M_{s}=1000,M_{1}=M_{2}=100$. More precisely, we have represented $\tau (1000,I_{1}\left(0\right),I_{2}\left(0\right))$ for $0\leq I_{1}\left(0\right),I_{2}\left(0\right)\leq 100$, also we have highlighted that τ(1000,49,51)≈0.3328.

On the other hand, in Table 4 we show the results of 10,000 trials of the Euler–Maruyama method with $▵t=10^{-4}$ and the value is represented in Figure 3. For each initial population size, we have written down the number of stops when S<1 or $I_{1}<1$ or $I_{2}<1$, and we have computed the mean stop time (mean) and its standard deviation (std). This result, which is close to the figure’s estimate, apparently differs from the deterministic solution, but not so much because, for example, with these initial values, the numerical solution is

$$S\left(0.8186\right)\;\approx \;0.0381,\;I_{1}\left(0.8186\right)\;\approx \;56.3229,\;I_{2}\left(0.8186\right)\;\approx \;2.8159,$$

and therefore it is not strange that a slight deviation or disturbance of the trajectory ends up at zero.

**Example** **3.** **A study of coexistence.***In this example, the per capital death and birth rates are*d(N)=1+5N/100 and b=6*. In addition,* $\beta_{1}=30,\beta_{2}=15,\alpha_{1}=4,\alpha_{2}=5/3$*, and* $b_{1}=12,b_{2}=6$*, so that* $R_{1}=2;R_{2}=1.5$*, and according Theorem 2 in [16] the solutions to deterministic model (8) converge to*

$$\lim_{t\to \infty }I_{1}\left(t\right)\;=\;15.13,\;\lim_{t\to \infty }I_{2}\left(t\right)\;=\;22.35.$$

**Figure 4:**
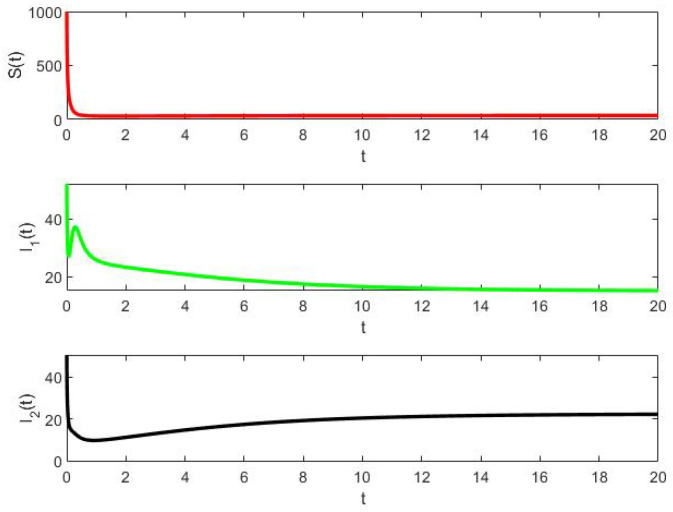
Example 3: deterministic solution for $S\left(0\right)=1000,I_{1}\left(0\right)=52,I_{2}\left(0\right)=51$.

In Figure 4, we have plotted the solution of (8) using Matlab for S(0)=1000, $I_{1}\left(0\right)=52$ and $I_{2}\left(0\right)=51$ in 0≤t≤20. The final solutions are S(20)≈35.7715, $I_{1}\left(20\right)\approx 15.1724$, and $I_{2}\left(30\right)\approx 22.1665$.

**Figure 5:**
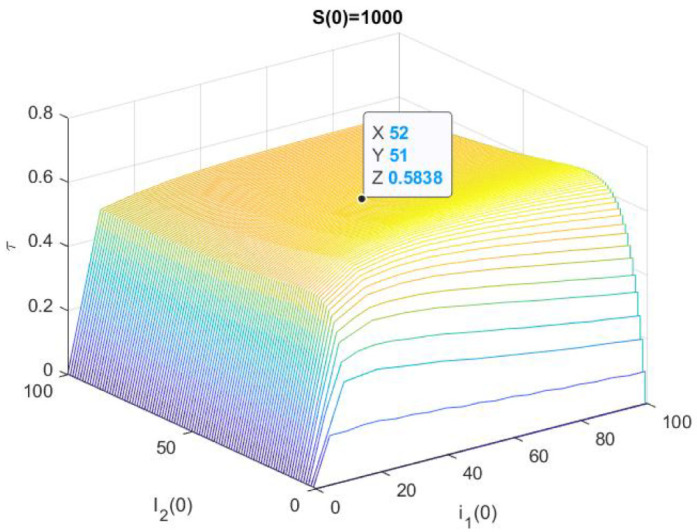
Example 3: S(0)=1000.

In Figure 5, we have represented the numerical solution of the problem (9) and (10) with $M_{s}=1000,M_{1}=M_{2}=100$. More precisely, we have represented $\tau (1000,I_{1}\left(0\right),I_{2}\left(0\right))$ for $0\leq I_{1}\left(0\right),I_{2}\left(0\right)\leq 100$, also we have highlighted that τ(1000,52,51)≈0.5838.

In Table 5, we show the results of 10,000 trials of the Euler–Maruyama method with $▵t=10^{-4}$ and the value representing in Figure 5. For each initial population size, we have written down the number of stops when S<1 or $I_{1}<1$ or $I_{2}<1$, and we have computed the mean stop time (mean) and its standard deviation (std). This result, which is close to the figure’s estimate, has no relation to the behavior of the deterministic system; in this example, the asymptotic behaviors of the two models are quite different.

## 4. Conclusions and Discution

In this paper, we have studied the coexistence of two pathogens model proposed in [16,17]. They showed that the deterministic and stochastic models differ considerably in prediction coexistence of the two pathogens because the probability of coexistence in the stochastic model is very small. We propose an alternative strategy to analyze the behavior of the stochastic model. More precisely, we have computed the mean persistence time for the stochastic model that satisfies a partial differential Kolmogorov type equation with degenerate coefficients. In order to do this, the finite element method has been used and the numerical implementation was performed using FreeFem++.

From our example we have showed the following.

1. Example 1: In this case, competitive exclusion occurs because in the deterministic model $\lim_{t\to \infty }I_{2}\left(t\right)=0$, while the stochastic model disappears somewhat more quickly.
2. Example 2: In this example, with vertical transmission of both strains, the deterministic solution cycles closer and closer while the stochastic solution is extinguished very quickly. The difference in the asymptotic behavior of deterministic and stochastic is very important.
3. Example 3: The difference with respect to the previous one is that now the first infection disappears as we can see in the Figure 5 and Table 5.

The main conclusion of this paper is evident: the deterministic and stochastic epidemic models differ considerably in predicting coexistence of the two diseases and in the extinction or not of one of them. Now, the main challenge would be to find an explanation for this result, in ([4], p. 3) we can read: “If the initial population size is small then a stochastic model is more appropriate, since the likelihood that the population becomes extinct due to chance must be considered.” Deterministic models often provide useful ways of gaining sufficient understanding about the dynamics of populations whenever they are large enough. This may be true in some cases but I propose the following analysis: in the elliptical differential Equation (2) (backward Kolmogorov equation) there exists two part, the advection with the vector μ and the diffusion with the matrix D, the first tends to move the solution while the second wears it down, in another words: which dominates, the advection or the diffusion? Let is define the ratio

(12) $$R\left(t\right)\;=\;\frac{||\;\mu \left(S\left(t\right),I_{1}\left(t\right),I_{2}\left(t\right)\right)\;{\left|\right|}_{2}}{||\;D\left(S\left(t\right),I_{1}\left(t\right),I_{2}\left(t\right)\right)\;{\left|\right|}_{2}},$$

comparing the evolution of these two terms of the equation.

**Figure 6:**
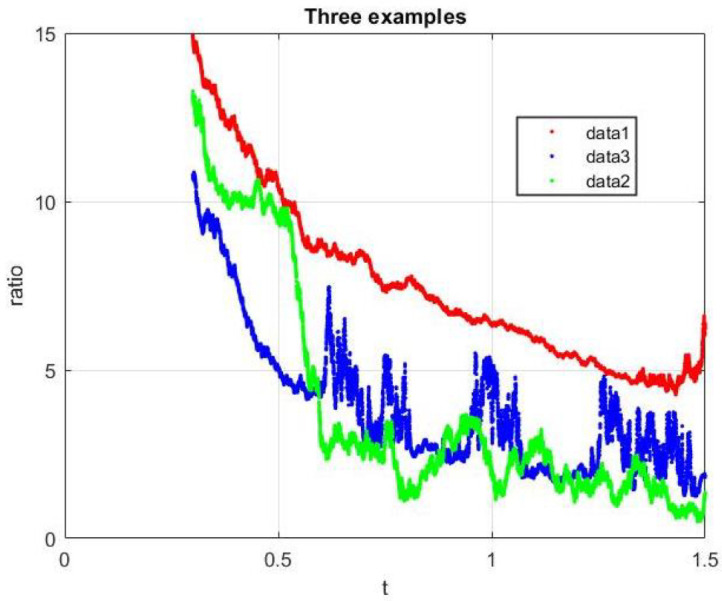
Evolution of the R(t) with Euler–Maruyama.

In Figure 6, we have plotted the evolution of $R_{j}$ using Euler–Maruyama in (7) with $▵t=10^{-4}$ and $S\left(0\right)=1000,I_{1}\left(0\right)=50$ and $I_{2}\left(0\right)=50$. Clearly the red trajectory (Example 1) decreases more slowly than the other two (Examples 2 and 3), this could explain its delay in extinction time. Obviously this does not prove anything, it only indicates a possible line of future research.

Finally, in my opinion we would need to make a previous estimate of the mean persistence time to fully understand the dynamics of a complete stochastic model. In my opinion, the stochastic model seems more realistic because, although it starts out swinging, it disappears reasonably after some time. The environment is constantly evolving, and as the philosopher Heraclitus wrote over 25 centuries ago: Everything changes and nothing remains still … and … you cannot step twice into the same stream.

## Figures and Tables

**Table 1:** Possible change in $X={\left(S,I_{1},I_{2}\right)}^{T}$ and their probabilities.

Changes	Probabilities
$▵X^{\left(1\right)}={\left[1,0,0\right]}^{T}$	$p_{1}=b\;S\;▵t$
$▵X^{\left(2\right)}={\left[0,1,0\right]}^{T}$	$p_{2}=b\;I_{1}\;▵t$
$▵X^{\left(3\right)}={\left[0,0,1\right]}^{T}$	$p_{3}=b\;I_{2}\;▵t$
$▵X^{\left(4\right)}={\left[-1,0,0\right]}^{T}$	$p_{4}=d\left(N\right)\;S\;▵t$
$▵X^{\left(5\right)}={\left[0,-1,0\right]}^{T}$	$p_{5}=\left(\alpha_{1}+d\left(N\right)\right)\;I_{1}\;▵t$
$▵X^{\left(6\right)}={\left[0,0,-1\right]}^{T}$	$p_{6}=\left(\alpha_{2}+d\left(N\right)\right)\;I_{2}\;▵t$
$▵X^{\left(7\right)}={\left[1,-1,0\right]}^{T}$	$p_{7}=b_{1}\;I_{1}\;▵t$
$▵X^{\left(8\right)}={\left[1,0,-1\right]}^{T}$	$p_{8}=b_{2}\;I_{2}\;▵t$
$▵X^{\left(9\right)}={\left[-1,1,0\right]}^{T}$	$p_{9}=\beta_{1}\;SI_{1}/N\;▵t$
$▵X^{\left(10\right)}={\left[-1,0,1\right]}^{T}$	$p_{10}=\beta_{2}\;SI_{2}/N\;▵t$
$▵X^{\left(11\right)}={\left[0,0,0\right]}^{T}$	$p_{11}=1-\sum_{i=1}^{10}p_{i}$

**Table 2:** Zeros of (8): each column corresponds to a zero.

Zeros	1	2	3	4	5	6
*S*	∗	∗	∗	0	∗	0
$I_{1}$	∗	0	0	∗	∗	∗
$I_{2}$	0	∗	∗	∗	∗	∗

**Table 3:** Example 1: Euler–Maruyama Method.

Initial Point		Number of Stops	Mean	Std
(1000, 50, 50)	S<1	0		
	$I_{1}<1$	120	1.6929	0.8579
	$I_{2}<1$	9880	2.5880	1.0005

**Table 4:** Example 2: Euler–Maruyama Method.

Initial Point		Number of Stops	Mean	Std
(1000, 49, 51)	S<1	4109	0.5716	0.0955
	$I_{1}<1$	14		
	$I_{2}<1$	5877	0.3276	0.1237

**Table 5:** Example 3: Euler–Maruyama Method.

Initial Point		Number of Stops	Mean	Std
(1000, 52, 51)	S<1	0		
	$I_{1}<1$	9543	0.9348	0.3894
	$I_{2}<1$	457	0.5024	0.3119

## Data Availability

None.
